# Soft Tissue Artefacts of the Human Back: Comparison of the Sagittal Curvature of the Spine Measured Using Skin Markers and an Open Upright MRI

**DOI:** 10.1371/journal.pone.0095426

**Published:** 2014-04-18

**Authors:** Roland Zemp, Renate List, Turgut Gülay, Jean Pierre Elsig, Jaroslav Naxera, William R. Taylor, Silvio Lorenzetti

**Affiliations:** 1 Institute for Biomechanics, ETH Zurich, Zurich, Switzerland; 2 Spine Surgery, Küsnacht, Switzerland; 3 Röntgeninstitut Zurich-Altstetten, Zurich, Switzerland; College of Basic Medical Science, Third Military Medical University, China

## Abstract

Soft tissue artefact affects the determination of skeletal kinematics. Thus, it is important to know the accuracy and limitations of kinematic parameters determined and modelled based on skin marker data. Here, the curvature angles, as well as the rotations of the lumbar and thoracic segments, of seven healthy subjects were determined in the sagittal plane using a skin marker set and compared to measurements taken in an open upright MRI scanner in order to understand the influence of soft tissue artefact at the back. The mean STA in the flexed compared to the extended positions were 10.2±6.1 mm (lumbar)/9.3±4.2 mm (thoracic) and 10.7±4.8 mm (lumbar)/9.2±4.9 mm (thoracic) respectively. A linear regression of the lumbar and thoracic curvatures between the marker-based measurements and MRI-based measurements resulted in coefficients of determination, R^2^, of 0.552 and 0.385 respectively. Skin marker measurements therefore allow for the assessment of changes in the lumbar and thoracic curvature angles, but the absolute values suffer from uncertainty. Nevertheless, this marker set appears to be suitable for quantifying lumbar and thoracic spinal changes between quasi-static whole body postural changes.

## Introduction

Back pain is an increasingly common affliction, with approximately one-third of the population suffering from low back pain at any given time [1]. Kinematic parameters of the lumbar spine, such as the rate of angular rotation and linear displacement at the joints (L3/L4; L4/L5; L5/S1), especially during the onset of lumbar flexion, are useful for discriminating between individuals with and without low back pain [2]. While motion of the lumbar spine is accessible using video fluoroscopy [2]–[4], the approach is highly invasive and exposes subjects to unnecessary X-ray radiation [4]. Moreover, while novel dynamic and non-invasive approaches now exist for assessing functional motion of the back, even over extended periods of time [5]–[8], the accuracy of such methods for evaluating the underlying skeletal kinematics remains unknown.

In addition to instability and degeneration of supporting soft tissue structures, overloading is considered a main cause of low back pain due to a combination of cumulative or acute loads [9], [10]. However, determination of the internal loading conditions requires knowledge of the spine's position and movement. While bone pins allow direct access to skeletal kinematics [11]–[13], they are only rarely used due to their invasive nature. Motion analysis, on the other hand, allows the non-invasive investigation of motion patterns [14]. However, while skin markers are easy to apply and rarely limit the subject's movement, they are affected by soft tissue artefact (STA), which results from motion of the skin relative to the underlying bones due to inertial effects, skin elasticity and deformation caused by muscle contraction [15]–[17]. STA occurs in all directions, and the distributions are known to be non-uniform [18].

To obtain an understanding of the accuracy of skin markers for assessing spinal kinematics, a marker set has been developed that allows global parameters of curvature angles [19]–[25] and back segment rotations [26]–[30] to be investigated [31]. However, the accuracy and precision of these analyses remain unknown. Several studies have validated skin markers on different body regions [18], [32]–[38], but validation studies of back markers are rare [26]–[28], [39], [40] (Table 1), and few have been validated against global spinal shape, including spinal segment curvature or rotations.

**Table 1:** Literature summary of the soft tissue artefact of different body locations.

Body location	Author	Soft tissue artefact	Motion
**Foot**	Tranberg and Karlsson [34]	Up to 4.3 mm	Static weight-bearing position
	Maslen and Ackland [35]	Mean marker error up to 14.9 mm	Static weight-bearing position
**Shank**	Gao and Zheng [18]	Inter-marker movement up to 9.3 mm	Level walking
	Garling et al. [36]	Up to 11 mm	Step up
	Sangeux et al. [32]	Up to 7 mm	Static non-weight-bearing; knee flexion between 0° and 90°
**Thigh**	Gao and Zheng [18]	Inter-marker movement up to 19.1 mm	Level walking
	Garling et al. [36]	Up to 17 mm	Step up
	Sangeux et al. [32]	Up to 22 mm	Static non-weight-bearing; knee flexion between 0° and 90°
	Akbarshahi et al. [38]	RMSE up to 29.3 mm around the knee joint	Functional activity: open-chain knee flexion, hip axial rotation, level walking, step up
**Scapula**	Matsui et al. [33]	Mean marker error of about 67 mm	Arm elevation
**Finger**	Ryu et al. [37]	Up to 10.9 mm	Hand flexion
**Back**	Morl and Blickhan [39]	Up to 9.86 mm at lumbar levels L3 and L4	Rotated seating: shoulder turned approximately 90° with respect to the pelvis
	Heneghan and Balanos [40]	Up to 16 mm at thoracic levels (T1, T6, T12)	35° of axial rotation in a seated upright position
		Up to 1.5 mm at thoracic levels (T1, T6, T12)	Single arm elevation in a seated upright position
**Trunk**	This study	Up to 27.4 mm	Static sitting position

Using open MRI and skin markers, the goal of this study was to determine the magnitude and direction of STA on the back and compare the spinal curvature and segment angles. We then examined whether the shape of the spine (lumbar and thoracic curvature angle and lumbar and thoracic segment rotation in the sagittal plane) can be measured with sufficient accuracy to determine spinal shape between posture changes or during quasi-static movements using skin markers.

## Methods

### Accuracy of the marker and vertebra positions using MRI

**Figure 1:**
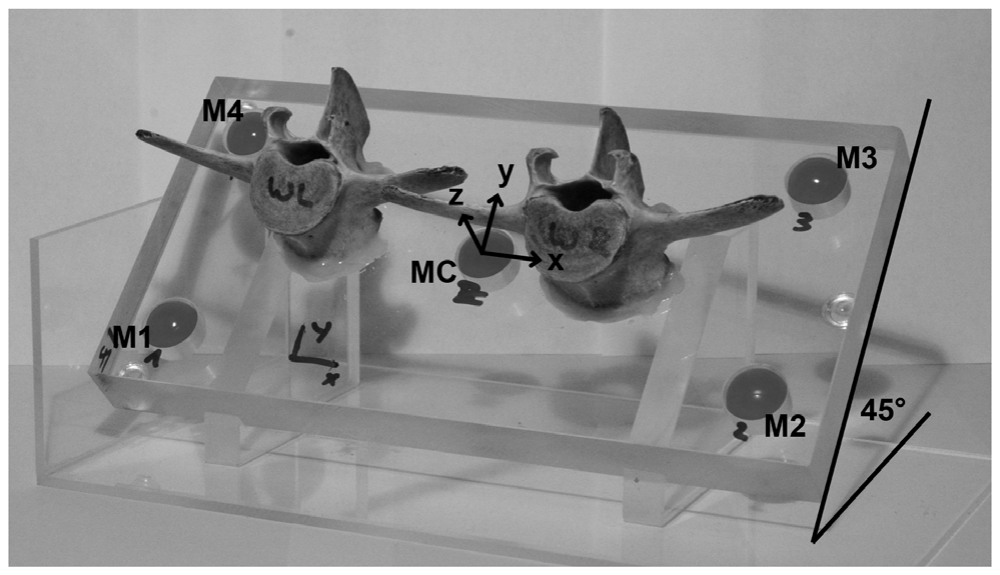
Acrylic glass plate with five MRI visible markers (paintballs: M1, M2, M3, M4, MC, with distances of M1–M2 = 180 mm, M2–M3 = 70 mm) and two lamb vertebrae, shown in the forward tilted position (45°).

The accuracy of assessing vertebral location using MRI was determined using a plate of acrylic glass with five MRI-visible skin markers (paintballs) and two lamb vertebrae (Figure 1). The paintballs were placed into precision-drilled holes (±0.1 mm) in each of the four corners and middle of the plate. The two vertebrae were glued onto the plate between the markers. The plate was then examined using an open MRI (Upright Multi-Position MRI; 0.6 Tesla; Fonar Corporation, Melville, USA) in horizontal (0°), forward tilted (45°) and vertical (90°) positions in order to quantify the accuracy of the marker and vertebrae locations as well as the orientation of each vertebrae base plate plane (BPP) relative to horizontal.

### Subjects

**Figure 2:**
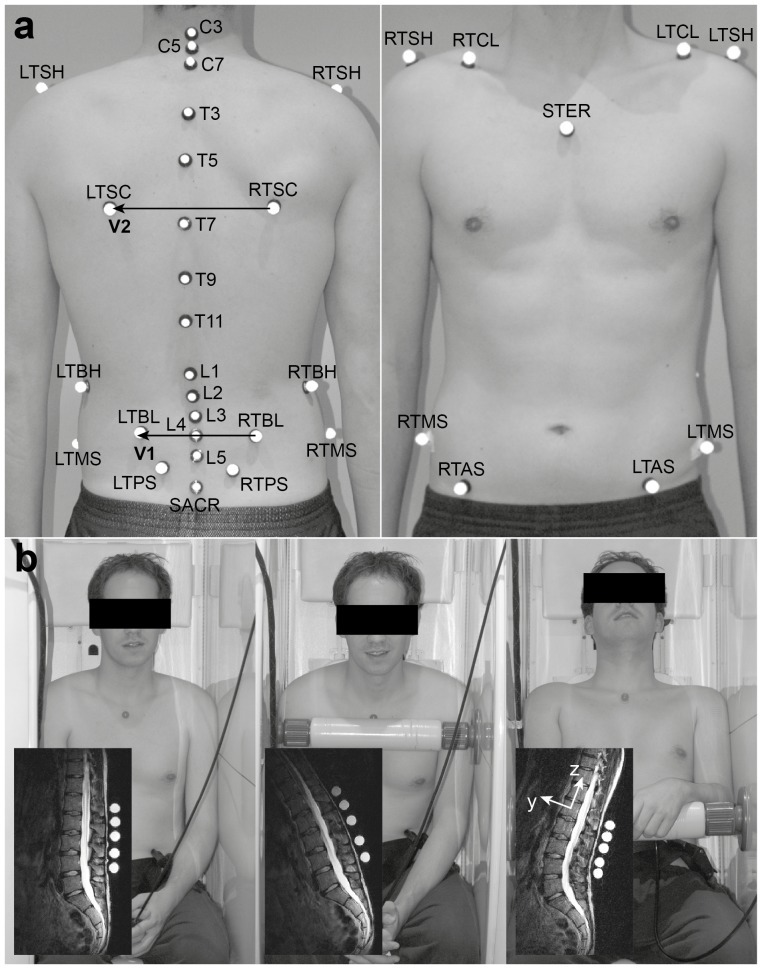
Measurement set-up including (a) the “IfB-marker-set” of the trunk and the pelvis (for explanation of abbreviations and for segmental allocation, see Table 2) and (b) the three analysed seating positions with an example of a corresponding MR image including a local coordinate system of a vertebral body. Upright seating position: lower spine had partial contact with the backrest, and the whole upper body was in an upright position. Flexed seating position: upper body was tilted about 30° forward and supported on a bar, while the arms were rested on their lap. Extended seating position: the subject's bottom was pushed approximately 20 cm forward and the head was supported by the backrest. (from left to right).

Seven healthy subjects (three female; average age 29 y (range 22–46); height 174 cm (160–184); mass 71 kg (55–96)) provided written informed consent to participate in this pilot study that was approved by the local ethics committee. Subject recruitment was achieved through voluntary participation after public poster advertising. The participant on Figure 2 has seen this manuscript and figure and has provided written informed consent for its use in publication. A wide range of subject height and weight was chosen in order to exemplarily investigate the range of kinematics that could be observed within a broad population. A power analysis (one-tailed paired t-test, α = 0.05, β = 0.1) performed using a statistical software package (G*Power 3.1.3) [41], based on the determined accuracy of the acrylic plate measuring system (SD of the curvature angle due to the measurement system: 3.6°) and one test measurement (difference in lumbar curvature angle between the upright and extended position: 8°) with an effect size *dz* of 1.571 (where 
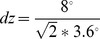
) revealed a minimum subject number of six with a power level of 0.948.

### Instrumentation

T2-weighted sagittal images were taken with a repetition time of 2750 ms, an echo time of 110 ms and a layer thickness of 4 mm. The resolution was 240×240 in an image plane of 360×360 mm, providing a voxel size of 1.5*1.5*4 mm^3^. The layers were ranked without any gaps between the marginal markers. As a consequence, the lumbar and thoracic regions required approximately 35 and 50 images respectively, corresponding to a measurement time of approximately seven minutes for each posture.

### Marker set

Based on our whole body “IfB-marker-set” [42]–[47] (Figure 2a), only the markers for the lumbar and thoracic segments were used (Table 2). The markers were MRI-visible commercial paintballs (BrassEagle Wild Streak Paintballs, diameter 17.3 mm), which consisted of a dyed liquid surrounded by a thin gelatin shell. After palpation, performed in an upright standing position, the markers were mounted on washers and were fixed to the skin using a toupee plaster. In order to provide support during sitting while preventing marker contact with the backrest, two foam tubes were attached to the paraspinal muscle bellies. The subjects' lumbar and thoracic spines were then measured in the MRI in upright, flexed and extended seating positions (Figure 2b).

**Table 2:** Marker placement, segment allocation and abbreviations.

Abbreviations	Marker placement	Segment allocation
RTSH, LTSH	Right and left acromion	Upper trunk segment
RTCL, LTCL	Right and left clavicula	
C7	7^th^ cervical vertebrae	
C3, C5	3^rd^, 5^th^ cervical vertebrae	
STER	Sternum	Thoracic segment
RTSC, LTSC	Right and left inferior angle of the scapula	
RTBH, LTBH	Right and left most inferior rib	
T3, T5, T7, T9, T11	3^rd^, 5^th^, 7^th^, 9^th^, 11^th^ thoracic vertebrae	
RTBL, LTBL	Right and left lateral back on height of L4	Lumbar Segment
L1, L2, L3, L4, L5	1^st^, 2^nd^, 3^rd^, 4^th^, 5^th^ lumbar vertebrae	
RTAS, LTAS	Right and left anterior superior iliac spine	Pelvic segment
RTPS, LTPS	Right and left posterior superior iliac spine	
RTMS, LTMS	Right and left mid superior iliac spine	
SACR	Sacrum	

### Data analysis

The MR images were manually segmented using Avizo (v5.1, Mercury Computer Systems Inc., Burlington, USA). Spheres were fitted to the markers (Geomagic Studio, v9, Raindrop Geomagic, USA), and the normal vector of each vertebral body's BPP and centre of gravity (CoG) were determined.

Data analysis was performed using MATLAB (vR2010a, MathWorks Inc., Natick, USA). STA was described by changes in the vectors pointing from the vertebral bodies' CoG to the corresponding marker. The normal vector of the BPP defined the cranial (z) axis of each vertebral body coordinate system. As an exception, due to increased segmentation stability, the z-axis of the fifth lumbar vertebra was determined using the upper plate, and rotated accordingly. The y-axis was the cross-product of the z-vector with the unit vector in the anterior-posterior direction of the MRI, and the x-axis was defined by the normalised cross-product of the y and z vectors (Figure 2b). Each local coordinate system was located at the CoG of the respective vertebral body. The vectors from the vertebral bodies' CoG to the corresponding markers were constructed using the local coordinate systems. The differences between this vector in the upright and the flexed or extended seating positions described the magnitude and direction of STA of each marker on the spinous process.

To determine the curvature angles [48] of the lumbar (***α*** *_lumbar_*) and thoracic (***α*** *_thoracic_*) spines, the sagittal planes of the spines were defined normal to the vectors from RTBL to LTBL (V_1_) and from RTSC to LTSC (V_2_) respectively (Figure 2a). The position vectors of the markers and the vertebral bodies were projected onto this plane. Circles [49] were created for the lumbar and thoracic spines that best fitted the CoGs of L1–L5, and T3, T5, T7, T9 & T11 respectively. ***α*** *_lumbar_* was then calculated as the angle between the two radius vectors from the circle centre to the CoG of L1 and L5, and ***α*** *_thoracic_* accordingly as the angle between the radius vectors T3 & T11. The same angles were calculated for the corresponding lumbar and thoracic markers. Kyphosis was defined as a positive angle (**α**>0).

To analyse the accuracy with which the skin markers were able to represent the rotation of the vertebral bodies, the mean sagittal rotation error (ESR) of the lumbar and thoracic segments (Table 2) was calculated. Marker cloud registration was performed using a least squares method^41^. The sagittal rotation of the lumbar and thoracic segments was calculated between the corresponding marker cloud in the upright and compared to the flexed or extended positions. Here, the rotation of each vertebral section was calculated using a 3D regression line, fitted through the vertebral CoGs, and compared against the rigid rotation of the relevant marker cloud.

Due to the large radius of the paintballs, some lumbar markers of subjects 3, 4, and 7 touched each other in the extended seating position and it was not possible to analyse these MR images. Owing to image blur as a result of body movement during the measurements, the thoracic MR images of subject 2 in the flexed seating position were not taken into account for the analysis.

### Statistics

All statistics were determined using IBM SPSS Statistics (v19, SPSS Inc., Chicago, USA). Statistical significance was defined as p<0.05. The absolute marker artefact was analysed using analyses of variance (ANOVA) for the subjects, positions and marker locations. Furthermore, correlations between the curvature angles based on the marker and vertebral body coordinates were investigated using linear regression analysis.

## Results

### Accuracy of the marker and vertebra positions using MRI

The accuracy of the MRI and the image segmentation was similar to that of a conventional motion capture system. Based on the mean error between different markers on the acrylic plate (0.6±0.5 mm) and between the vertebral CoGs and the markers (1.1±1.1 mm), the direction-related measurement uncertainties (σ_x_, σ_y_, σ_z_) of the markers were (1.0 mm, 0.5 mm, 0.5 mm) and for the vertebral bodies (2.0 mm, 1.0 mm, 1.0 mm). The orientation of the BPP relative to horizontal varied by up to 2.7°. The mean error was 1.6±1.2°.

### Subject measurements

The mean STA in the flexed and extended positions were 10.2±6.1 mm (lumbar)/9.3±4.2 mm (thoracic) and 10.7±4.8 mm/9.2±4.9 mm respectively. The largest STA was 27.4 mm for marker SPL5 in the flexed position. The STA was significantly different between subjects (p<0.001 lumbar and thoracic), but no differences were observed for either markers (p = 0.604 lumbar, p = 0.404 thoracic) or seating positions (p = 0.428 lumbar, p = 0.926 thoracic) (Table 3). The subject's mean STA of the lumbar and thoracic markers as well as the flexed and extended positions varied between 6.2 mm and 13.2 mm for the seven subjects with a BMI between 20.6 kg/m^2^ and 30.3 kg/m^2^. However, no clear relationship between STA and BMI was observed.

**Table 3:** Direction-related (r_x_, r_y_, r_z_) mean marker artefact (mean) and the absolute values (|r|) with their standard deviations (SD) of the lumbar and thoracic skin markers in the flexed and extended positions.

		Flexion [mm]			
	Marker	r_x_ (mean (SD))	r_y_ (mean (SD))	r_z_ (mean (SD))	|r| (mean (SD))
**Thoracic**	**SPT1 (T3)**	0.6 (1.8)	0.1 (1.3)	5.1 (2.3)	5.4 (2.0)
	**SPT2 (T5)**	0.0 (4.4)	0.1 (2.3)	−3.1 (4.8)	6.7 (2.4)
	**SPT3 (T7)**	0.4 (3.7)	1.4 (2.3)	−8.0 (4.8)	8.9 (5.0)
	**SPT4 (T9)**	2.9 (4.5)	2.2 (4.2)	−10.4 (1.4)	12.2 (2.7)
	**SPT5 (T11)**	0.1 (4.8)	1.7 (6.6)	−7.2 (5.1)	10.7 (4.6)
**Lumbar**	**SPL1 (L1)**	−1.8 (2.7)	−1.7 (2.7)	−7.1 (3.4)	8.5 (3.0)
	**SPL2 (L2)**	−2.6 (3.6)	−1.8 (2.2)	−6.2 (5.2)	8.4 (4.4)
	**SPL3 (L3)**	−3.7 (4.0)	−1.7 (2.9)	−7.1 (7.6)	10.1 (6.4)
	**SPL4 (L4)**	−3.9 (4.8)	−2.6 (2.7)	−6.2 (9.6)	10.7 (7.7)
	**SPL5 (L5)**	−4.1 (5.6)	−2.6 (3.8)	−9.1 (10.0)	13.2 (8.2)

		Extension [mm]			
	Marker	r_x_ (mean (SD))	r_y_ (mean (SD))	r_z_ (mean (SD))	|r| (mean (SD))
**Thoracic**	**SPT1 (T3)**	2.0 (1.6)	2.2 (3.4)	3.4 (9.1)	8.0 (6.4)
	**SPT2 (T5)**	−0.6 (3.1)	0.7 (4.9)	2.9 (9.4)	9.3 (5.7)
	**SPT3 (T7)**	0.2 (6.0)	−3.3 (3.9)	−2.3 (8.9)	10.3 (5.2)
	**SPT4 (T9)**	2.7 (4.9)	0.8 (2.4)	−5.7 (7.9)	10.0 (5.0)
	**SPT5 (T11)**	−0.1 (4.3)	1.4 (2.4)	−4.1 (6.4)	8.0 (3.7)
**Lumbar**	**SPL1 (L1)**	−1.1 (2.3)	0.8 (1.4)	10.3 (6.3)	10.6 (6.3)
	**SPL2 (L2)**	−2.2 (2.1)	2.7 (2.6)	8.5 (5.8)	10.1 (4.6)
	**SPL3 (L3)**	−2.8 (3.3)	2.9 (7.3)	8.2 (7.6)	12.1 (6.2)
	**SPL4 (L4)**	−3.8 (2.1)	2.4 (2.6)	9.5 (7.7)	11.8 (5.8)
	**SPL5 (L5)**	−4.8 (2.0)	3.7 (2.9)	1.0 (6.4)	8.8 (1.1)

**Figure 3:**
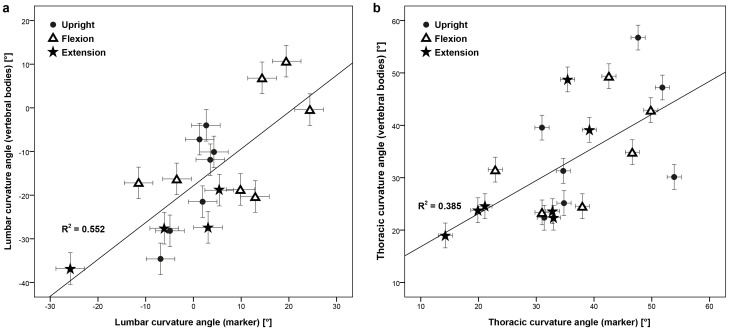
Scatter diagram of the lumbar (a) and thoracic curvature angle (b) in the upright (circle), flexed (triangle) and extended positions (star). The x-axis represents the values calculated from the markers, and the y-axis from the vertebral bodies. The crosses show the open MRI's measurement uncertainty of the curvature angles calculated from the markers (x-axis) and calculated from the vertebral bodies (y-axis).

**Figure 4:**
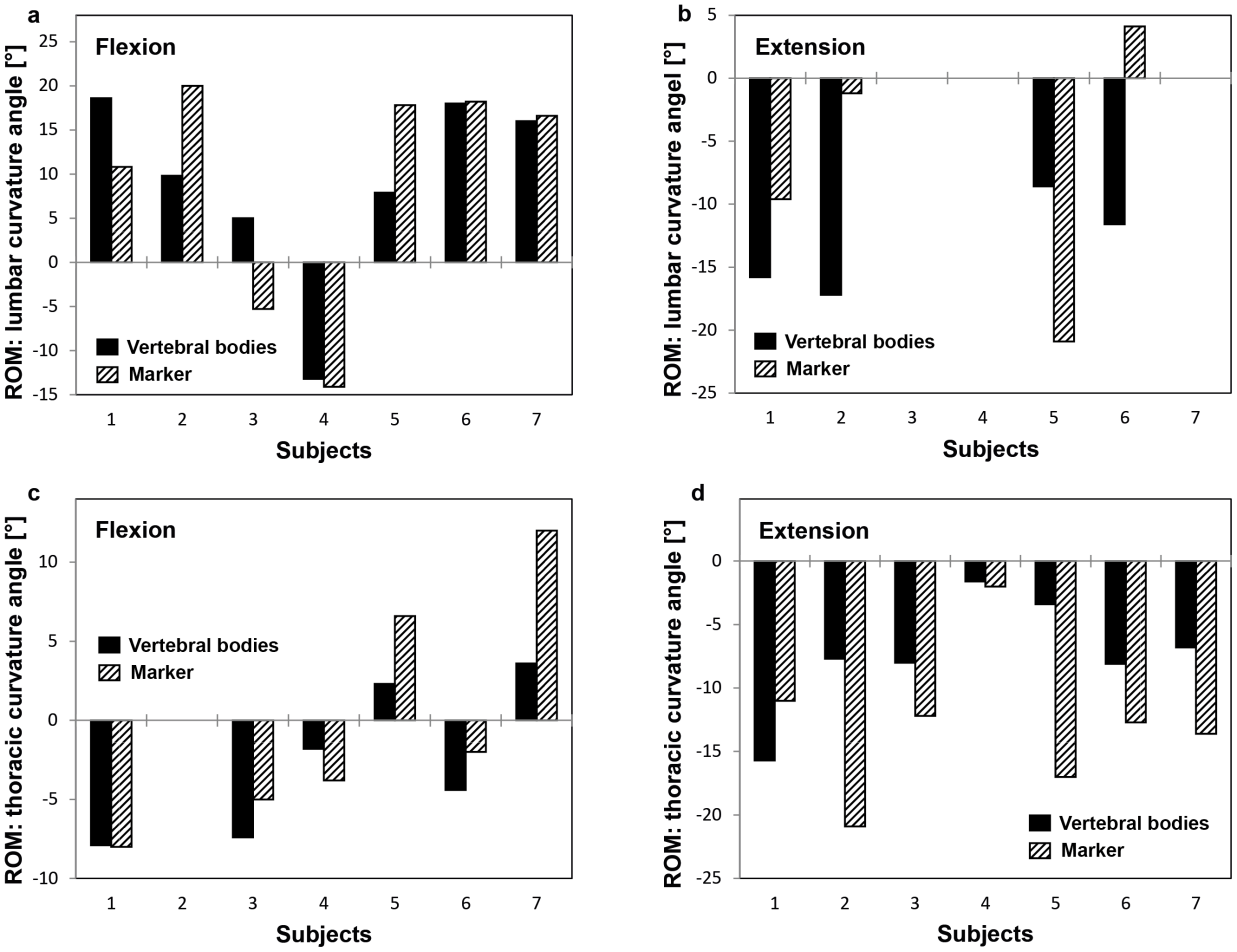
Range of lumbar (a/b) and thoracic (c/d) curvature angle of the subjects, calculated using the vertebral bodies (black) and the skin markers (hatched). The range was defined from the upright to the flexed (a/c) and to the extended positions (c/d).

The lumbar (***α*** *_lumbar_*) and thoracic curvature angles (***α*** *_thoracic_*) calculated using the markers and the vertebral bodies revealed no clear correlation (R^2^ = 0.552 (lumbar); R^2^ = 0.385 (thoracic); Figure 3). The root mean square errors (RMSEs) of the differences between the curvature angles determined by the markers and by the vertebral bodies were approximately two times higher for the lumbar spine than the thoracic spine (Table 4). The lumbar curvature angles from the upright to the flexed and extended positions showed the same sign in six of the seven subjects and three of the four subjects respectively, whereas the sign of the thoracic curvature angle was the same in all subjects (Figure 4).

**Table 4:** Mean (SD) lumbar (***α*** *_lumbar_*) and thoracic curvature angle (***α*** *_thoracic_*) in the upright, flexed and extended sitting positions calculated with the vertebral bodies and the skin marker, as well as the mean differences (SD; RMSE) between the values from the skin marker and the vertebral bodies.

	Upright		Flexion		Extension	
	Vertebral bodies	Marker	Vertebral bodies	Marker	Vertebral bodies	Marker
	Difference		Difference		Difference	
***α_lumbar_*** ** [°]**	−16.8 (11.5)	0.3 (4.3)	−7.9 (13.2)	9.4 (12.6)	−27.7 (7.3)	−5.9 (14.2)
	17.1 (8.0; 18.6)		17.3 (11.2; 20.2)		21.8 (8.1; 22.9)	
***α_thoracic_*** ** [°]**	36.0 (12.4)	40.7 (10.0)	34.4 (10.2)	38.5 (10.1)	28.7 (10.9)	27.9 (9.4)
	4.7 (11.4; 11.5)		4.0 (9.4; 9.5)		−0.8 (8.3; 7.8)	

The ESR of the lumbar and thoracic segments calculated using the skin markers were 2.5±2.7° (RMSE: 3.6°) and −1.1±2.9° (RMSE: 3.0°) respectively. The largest ESRs were 6.6° (lumbar) and 9.1° (thoracic).

## Discussion

While the exact motion of the vertebrae remains unclear without the use of invasive approaches, knowledge on the accuracy of skin markers for assessing skeletal kinematics provides a baseline for identifying situations where non-invasive approaches are appropriate, and where not [50]. The measurement uncertainty of our MRI-based measurement system was similar to those of a typical motion capture system (1.5 mm) [51], with out-of-plane error about double the in-plane error. Analysis of the back STA produced similar results to those observed in other studies [39], [40] and also for other body parts (Table 1). The observed intra- and inter-individual patterns of STA during flexion and extension did not allow the determination of a common correction method by which to estimate the behaviour of single markers. In general, the inter-individual differences were larger than the differences within a single subject - a result that is in agreement with results from studies on the knee joint [36]. Knowledge of the influence of STA when using skin markers is required to ensure compensation for the largest error sources [16], [52], [53].

The present study only allowed the quantification of STA in a static set-up. However, we must be conscious of the fact that the spinal movement differs from other skeletal joints e.g. the hip or the knee, since the spine consists of several segments that allow movement relative to one another, including a different range of motion (ROM) in several planes. The present study only allowed the quantification of STA in a static set-up. It must be assumed that during dynamic activities, and especially impact situations, STA is even larger. For example, Akbarshahi and co-workers [38] found much larger marker STA during functional activities than Sangeux et al. [32], who used a static set-up (Table 1). Therefore, studies using static measurements seem to underestimate the magnitude of STA, possibly by a factor of two or more.

Due to the fact that the power analysis in this study was based on measurements using lamb vertebrae, the actual study in humans may require additional subjects to ensure sufficient power, due to secondary errors such as unintentional movements during MRI scanning. However, as a result of this power analysis, relatively few subjects were recruited into this study, and it was not possible to observe any clear relationships between STA and e.g. age, gender, height or material properties of the soft tissues. It is, of course, entirely possible that these or other subject-specific factors contribute to the magnitude of STA on the back. Here, it is quite conceivable for example that the individual elastic properties of the soft tissues have a strong influence on the magnitude of STA; such observations have been reported at other regions of the body (thigh) by Kratzenstein and co-workers [52], who also demonstrated locally varying STA, which was mainly attributed to muscle contraction and skin elasticity. In addition, no relationship between the levels of STA and BMI were observed in our study, but this could also be an artefact of the low number of subjects in our cohort. However, this finding is consistent with studies that used fluoroscopy [36] or bone pins [54] to examine the role of soft tissues on the underlying skeletal kinematics. Increasing the number of subjects could allow a better understanding of the relationships between BMI, age, gender, height or material properties of the soft tissues, but this was not the focus of the current study. In order to establish the influence of such subject-specific factors on STA, further research would be required in specific homogenous cohorts.

Since the correlations between the spinal curvature calculated from the skin markers and the vertebral bodies for each position (upright, flexion, extension) as well as for all positions together were low (Figure 3: R^2^ = 0.552 (lumbar), R^2^ = 0.385 (thoracic)), results that examine the lumbar and thoracic curvature angles by means of skin markers should be interpreted cautiously. This was possibly due to the fact that the anatomical distance and the material properties of the musculoskeletal tissues between the markers and CoG are generally not constant, resulting in inhomogenous deformation between the different positions. However, the range of curvature angles exhibited the same sign when comparing the upright with the flexed and the extended seating positions in 22 out of the 24 cases (Figure 4). Due to the fact that the range of curvature calculated from the skin markers did not consistently over- or underestimate that calculated from the vertebral bodies within subjects, positions or spinal segment, there is no clear method to enhance the accuracy of skin marker estimations through automated correction.

To summarise, the results of our study indicate that a change of lordotic/kyphotic shape, but not of the absolute amount of curvature, can be estimated using skin markers. Based on these findings, the use of the presented back marker set for analysing spinal motion seems to be as accurate as estimations of skeletal kinematics in the lower extremities (Table 1). Changes of the lumbar and thoracic curvature angle are measurable in the sagittal plane using the presented marker set, but measurement of the absolute curvature angles appears to be limited when using skin markers. These limitations associated with STA must be taken into account during non-invasive assessment of back motion before an improved understanding of the kinematics of subjects with and without back pain can be gained.
